# Suicidal Ideation in Major Depressed Individuals: Role of Type D Personality

**DOI:** 10.3390/jcm11226611

**Published:** 2022-11-08

**Authors:** Mokhtar Abdelhakim Laoufi, Benjamin Wacquier, Tristan Lartigolle, Gwenolé Loas, Matthieu Hein

**Affiliations:** Department of Psychiatry and Sleep Laboratory, Erasme Hospital, Université Libre de Bruxelles (ULB), 1070 Brussels, Belgium

**Keywords:** suicidal ideation, major depression, type D personality, risk factor

## Abstract

Major depressed individuals are a subpopulation at high-risk of suicide. However, despite the evidence for a particular relationship between suicidal ideation (SI) and type D personality, few studies have investigated the role played by this personality structure in the occurrence of SI in major depressed individuals. Data from 318 major depressed individuals recruited from the clinical database of the Sleep Laboratory were analysed. Suicidal ideation was considered present if the score in item 9 of the Beck Depression Inventory (BDI-II) was ≥1 and/or if they were highlighted during the systematic psychiatric assessment conducted on admission to the Sleep Laboratory. Logistic regression analyses were used to determine the risk of SI associated with type D personality in major depressed individuals. The prevalence of suicidal ideation was 38.4% in our sample of major depressed individuals. After adjusting for major confounding factors, multivariate logistic regression analyses demonstrated that type D personality was a risk factor for SI in major depressed individuals. Thus, given the potential role played by type D personality in the occurrence of SI in major depressed individuals, it seems necessary to more systematically research and adequately manage this personality structure to allow for a better prevention of suicidal behaviours in this subpopulation.

## 1. Introduction

Despite the different prevention strategies recommended by the World Health Organization, suicide remains an important public health issue worldwide. Indeed, the annual mortality by suicide is estimated at 800.000 individuals, and suicide is currently the second leading cause of death among young adults (<30 years) [1]. Moreover, this matter seems to have worsened following the negative psychological consequences of the COVID-19 pandemic [2]. Nevertheless, regardless of this negative impact of COVID-19, psychiatric disorders (such as major depression) remain the main risk factors for suicide in the general population [3]. In major depressed individuals with suicidal behaviours, the suicidal plan is generally associated with severe major depressive episodes characterised by the presence of chronic suicidal ideation (SI) [4,5]. However, in major depressed individuals, SI is a frequent symptom (37.7%) and may lead to suicidal behaviours in 15% of cases [6,7]. In this particular subpopulation, therefore, SI seems to play a major role in suicide risk since it constitutes the first step for major depressed individuals in their suicidal plan [8]. Given these elements, additional investigations seem to be necessary to identify the specific factors associated with the occurrence of SI in major depressed individuals to allow for a better prevention of suicidal behaviours in this particular subpopulation.

Type D personality is a stable personality structure characterised by negative affectivity and social inhibition [9]. In the literature, evidence in favour of a particular relationship between type D personality and SI exist [10]. Indeed, the prevalence of type D personality may reach 51.6% in individuals with SI, whereas suicidality scores are higher in individuals with type D personality [11,12]. In addition, type D personality appears to be a risk factor for SI in both the general population and some subpopulations [12,13]. However, despite a non-negligible prevalence of type D personality in major depressed individuals [14], few studies have investigated the potential involvement of this personality structure in the occurrence of SI in this particular subpopulation [15]. Thus, it could be interesting to study the risk of SI associated with type D personality in major depressed individuals in order to allow for a better understanding of the higher suicidality in this specific subpopulation.

The aim of this study was therefore to empirically investigate the risk of SI associated with type D personality in a large sample of major depressed individuals. Our hypothesis was that type D personality plays a major role in the occurrence of SI in major depressed individuals. The objective of this approach was to provide healthcare professionals caring for major depressed individuals with reliable data regarding the risk of SI associated with type D personality in order to allow for a better identification of major depressed individuals at high risk of SI and the establishment of more effective suicide prevention strategies in this particular subpopulation.

## 2. Materials and Methods

The methodology used in this study is similar to that used in previous studies of our research group [16,17].

### 2.1. Population

**Figure 1:**
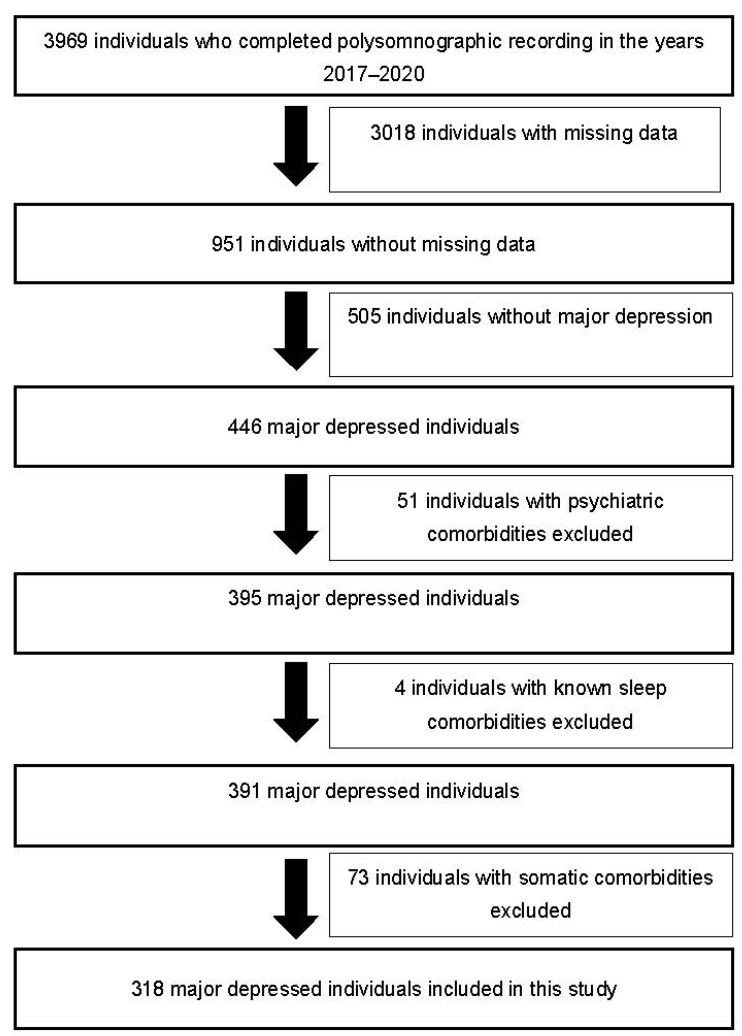
Selection diagram of major depressed individuals included in this study.

A total of 318 major depressed individuals were recruited from the database of the Erasme Hospital Sleep Laboratory, which contains clinical data from 3969 individuals who performed a polysomnographic recording between 2017 and 2020 (Figure 1). In our study, we did not recruit individuals without major depression because our objective was to focus on the subpopulation of major depressed individuals wherein the co-occurrence of type D personality may have a deleterious impact on psychiatric outcome [15].

These major depressed individuals were referred to the Sleep Laboratory by physicians specialised in sleep medicine after an outpatient consultation during which a preliminary assessment of their complaints related to sleep, their ongoing psychotropic/somatic treatments and their somatic/psychiatric comorbidities was systematically carried out in order to allow for a first diagnostic hypothesis. These patients were subjected to a polysomnographic recording to allow for an objective assessment of their sleep complaints and exclude the presence of comorbid sleep disorders negatively impacting mood regulation.

The inclusion criteria were age ≥18 years and the presence of a major depressive episode according to the DSM 5 diagnostic criteria [8].

The exclusion criteria were the presence of psychiatric disorders other than major depression, the presence of active SI with high-risk of suicidal behaviours during the stay in the sleep laboratory, the presence of current severe uncontrolled somatic pathologies (chronic liver pathologies, chronic pancreatic pathologies, chronic pulmonary pathologies, severe cardiovascular pathologies, severe renal pathologies, autoimmune pathologies, severe endocrine pathologies, severe neurological pathologies and pathologies altering the activity of the hypothalamic-pituitary-adrenal axis such as Cushing’s syndrome), the presence of current infectious diseases, the presence of active inflammatory diseases, the presence or history of head trauma with neurological sequelae, the presence or history of central nervous system damage that may affect the respiratory centres, the presence of craniofacial or thoracic malformations, the presence of ongoing pregnancy, the presence of predominantly central sleep apnoea syndrome, the presence of central hypersomnia, the presence of current parasomnia and the presence or history of drug addiction.

### 2.2. Method

In order to systematically diagnose their potential somatic pathologies, all major depressed individuals included in this study benefited during their admission to the Sleep Laboratory from a review of their medical records and a complete somatic assessment (including blood test, electrocardiogram, day electroencephalogram and urinalysis).

Afterwards, a complete psychiatric assessment was carried out by a unit psychiatrist in all major depressed individuals recruited in this study in order to allow for a systematic diagnosis of their potential psychiatric comorbidities according to the DSM 5 diagnostic criteria [8]. In addition, all major depressed individuals included in this study completed a series of self-administered questionnaires to assess the severity of their subjective complaints of depression (Beck Depression Inventory (BDI-II)), anxiety (Spielberger Anxiety Inventory), insomnia (Insomnia Severity Index) and daytime sleepiness (Epworth Sleepiness Scale) (the description of these self-questionnaires is available in Annex S1) [18,19,20,21]. Regarding the type D personality, it was assessed with the type-D scale (DS14). This scale consists of 14 items that may be scored from 0 to 4. It is subdivided into 2 subscales of 7 items: a negative affectivity scale and a social inhibition scale. A score ≥10 on each subscale indicates the presence of type D personality [22]. Following these different assessments, it was therefore possible to determine the presence or absence of SI in the major depressed individuals recruited in this study. Indeed, SI was considered present if the score in item 9 of the Beck Depression Inventory (BDI-II) [18] was ≥1 and/or if they were highlighted during the systematic psychiatric assessment.

Finally, all major depressed individuals included in this study benefited from a semi-structured sleep interview and a polysomnographic recording in order to systematically diagnose their potential comorbid sleep disorders according to the diagnostic criteria of the American Academy of Sleep Medicine (the description of this sleep assessment is available in Annex S2) [23].

### 2.3. Statistical Analyses

Statistical analyses were performed using Stata 14. The normal distribution of the data was verified using histograms, boxplots and quantile-quantile plots, and the equality of variances was checked using the Levene’s test.

In order to facilitate our analyses, we divided our sample of major depressed individuals into a control group without SI and a patient group with SI. Only the subjects with SI highlighted in the Beck Depression Inventory (BDI-II) (item 9) [18] and/or during the systematic psychiatric assessment during admission to the Sleep Laboratory were included in the “SI” group.

Given the asymmetric distribution of most continuous variables, non-parametric tests (Wilcoxon test) based on the medians (P25–P75) were used to demonstrate significant differences between the different groups of major depressed individuals. Regarding categorical variables, percentages were used for descriptive analyses and Chi^2^ tests were used for comparative analyses.

Univariate logistic regression models were used to study the risk of SI associated with type D personality and potential confounding factors (the detailed description of these confounding factors is available in Annex S3). In multivariate logistic regression models, the risk of SI associated with type D personality was only adjusted for significant confounding factors during univariate analyses. These different confounding factors were introduced hierarchically into the different multivariate models.

The adequacy of these different models was verified by the Hosmer and Lemeshow test, whereas the specificity of the model was verified by the Link test.

The results were considered significant when the *p*-value was <0.05.

## 3. Results

### 3.1. Demographic Data

Type D personality was present in 55.3% (*n =* 176) of the major depressed individuals from our sample (Table 1). Age <30 years, age >45 years, use of benzodiazepine receptor agonists, antidepressant therapy, presence of trait anxiety alone, presence of trait + state anxiety, Beck Depression Inventory (BDI-II) (reduced to 20 items) scores ≥21 and type D personality were more frequent in major depressed individuals with SI than in major depressed individuals without SI (Table 1). In addition, compared to major depressed individuals without SI, major depressed individuals with SI had higher scores on the Beck Depression Inventory (BDI-II), the Beck Depression Inventory (BDI-II) (reduced to 20 items), the Spielberger Anxiety Inventory—Trait, the Spielberger Anxiety Inventory—State, the type-D scale (DS14), the type-D scale (DS14)—Negative Affectivity subscale and the type-D scale (DS14)—Social Inhibition subscale (Table 1). The two groups of major depressed individuals did not differ significantly for the other demographic data. Finally, in major depressed individuals, SI was very frequent since its prevalence was 38.4% (*n =* 122) in this particular subpopulation (Table 1).

### 3.2. Univariate Analyses

Age <30 years, age >45 years, use of benzodiazepine receptor agonists, antidepressant therapy, presence of trait anxiety alone, presence of trait + state anxiety, Beck Depression Inventory (BDI-II) (reduced to 20 items) scores ≥21 and type D personality were associated with a higher risk of SI in major depressed individuals (Table 2).

### 3.3. Multivariate Regression Analyses

After adjusting for the main confounding factors highlighted during the univariate analyses, multivariate analyses demonstrated that type D personality was a risk factor for SI in major depressed individuals (OR 1.76 (95% CI 1.02 to 3.01), *p*-value = 0.041) (Table 3).

### 3.4. Polysomnographic Data

There was no significant difference between major depressed individuals without SI and major depressed individuals with SI for the different polysomnographic parameters (Table 4).

## 4. Discussion

The prevalence of SI highlighted in our study (38.4%) seems to be higher than that of other studies investigating the specific relationship between type D personality and SI. Indeed, the prevalence of SI was 7.5% for the study by Michal et al. (2010) [11] and 20.0% for the study by Walters et al. (2018) [24]. However, unlike our study wherein the main inclusion criterion was the presence of a major depressive episode, the study by Michal et al. (2010) [11] focused on the general population, whereas the study by Walters et al. (2018) [24] only recruited individuals with atrial fibrillation. The differences in the recruited populations could explain the higher prevalence of SI in our study since major depressive episodes are one of the main risk factors for SI [25]. On the other hand, the prevalence of SI in major depressed individuals demonstrated in our study (38.4%) seems to be consistent with that of the meta-analysis by Cai et al. (2021) (37.7%) [6], which highlights the importance of this psychiatric symptom in this particular subpopulation. Thus, in our study, we have demonstrated that, similar to the data available in the literature [16,17], major depressed individuals referred for polysomnographic recordings are a subpopulation at high risk of SI, which seems to justify a better screening and adequate management of this psychiatric symptom in this particular subpopulation.

In agreement with the literature [14,15], we demonstrated that the prevalence of type D personality is high (55.3%) in major depressed individuals. In addition, we have shown that type D personality was a risk factor for SI in major depressed individuals, which seems to be consistent with the limited data available [15]. Pathophysiologically, several elements could allow for a better understanding of this high prevalence of type D personality and its association with SI in major depressed individuals. First, type D personality appears to be a vulnerability factor for the development of major depressive episodes [14,26,27]. Indeed, some biological (alterations of the hypothalamic–pituitary–adrenal axis and oxidative stress) and behavioural (unhealthy lifestyle) mechanisms associated with type D personality play a central role in the pathophysiology of major depression [28,29,30,31,32,33]. However, the presence of this higher vulnerability to major depressive episodes in individuals with type D personality could explain the high prevalence of this personality structure in our sample of major depressed individuals. Second, in individuals with personality type D, there is major psychological pain induced by some intrapsychic (depression, alcohol abuse and post-traumatic stress) and interpersonal (low belonging, social isolation, lack of support) vulnerability factors [10,34]. In addition, the avoidance tendency of individuals with type D personality may reinforce this psychological pain following the occurrence of a recurrent feeling of helplessness induced by persistent difficulties in solving problems [10,34]. However, in order to avoid this major psychological pain, some individuals with type D personality may develop dysfunctional coping strategies characterised by the occurrence of SI [10,34], which could explain the higher risk of SI associated with this personality structure in major depressed individuals demonstrated in our study. Thus, given these different elements, it seems necessary to systematically screen and adequately manage the type D personality in major depressed individuals in order to allow for a better prevention of suicidal behaviours in this particular subpopulation.

Therapeutically, the demonstration of this higher risk of SI associated with type D personality could allow for the development of new strategies for the management of this psychiatric symptom in major depressed individuals with this personality structure. Indeed, in order to reduce the major psychological pain present in individuals with type D personality, several therapeutic strategies may currently be used to improve their mood (cognitive–behavioural therapy, mindfulness-based cognitive training, relaxation therapy and pharmacotherapy), their lifestyle habits (exercise activity, smoking cessation, compliance with medical regimen and compliance with lifestyle advice) and their interpersonal functioning (interpersonal therapy, assertiveness training and reinforcement of appropriate health care seeking) [35,36]. However, given the central role played by psychological pain in the occurrence of SI [37], the implementation of these therapeutic strategies could potentially reduce the frequency and severity of SI in major depressed individuals with type D personality through a better ability to adapt to stress factors, a reduction in negative affectivity and better psychosocial integration [35,36,38,39]. Nevertheless, despite the potential beneficial effect on SI of these therapeutic strategies that may reduce the major psychological pain induced by type D personality, it is essential to establish adequate treatment for major depression in order to allow for an integrated management of SI in major depressed individuals with type D personality [40].

In our univariate analyses, we found that alongside type D personality, other factors seem to be associated with the occurrence of SI in our sample of major depressed individuals. Indeed, similar to the literature [41], we demonstrated that the depression severity and the presence of anxiety symptoms play a central role in the development of SI for major depressed individuals, which could be explained by several factors. First, since SI is a severity marker of major depressive disorder, it generally occurs more frequently in individuals with more severe major depressive episodes [42]. Second, in major depressed individuals, anxiety symptoms may promote the development of SI given their negative impact on both life quality and clinical outcome [43]. On the other hand, in our study, major depressed individuals treated with antidepressants and benzodiazepine receptor agonists seem to present higher risks of SI. However, it is important not to invert the causal links based on these results. Indeed, major depressed individuals with SI are more frequently treated with antidepressants and benzodiazepine receptor agonists because they usually have more severe forms of major depression requiring psychotropic treatment [44]. Finally, in our study, young age (<30 years) and middle-to-old age (>45 years) seem to have an impact on the occurrence of SI in major depressed individuals. Although young age is a demonstrated risk factor for SI in major depressed individuals, the prevalence of SI tends to be lower in older populations [45,46]. This difference compared to the literature could possibly be explained by the fact that compared to other studies wherein SI is generally only evaluated by self-questionnaires [47], SI was assessed by a systematic psychiatric assessment combined with self-questionnaires in our study, which may have allowed for a better evaluation of SI in older major depressed individuals who tend to minimise their SI during self-questionnaires [48]. Thus, in major depressed individuals, it seems important to take into account these psychiatric (depression severity and anxiety symptoms) and demographic (age) factors in order to allow for an optimal assessment of the risk of suicidal behaviours in this particular subpopulation.

### Limitations

The results obtained in our study come from retrospective data which, even if they have been encoded in a systematic manner, cannot be verified directly with the subjects in most cases. Our results thus need to be replicated in prospective studies. Moreover, we focused only on type D personality, which means that our results cannot be generalised to other personality structures. In addition, since our main inclusion criterion was the presence of a major depressive episode, our results are not applicable to other psychiatric disorders, which may limit their interpretation. Finally, our database only contains major depressed individuals who have agreed to perform a polysomnographic recording, which may also limit the generalisation of our results.

## 5. Conclusions

In our study, we confirmed that SI was frequent in major depressed individuals. In addition, we have shown that type D personality was a risk factor for SI in major depressed individuals, which seems to justify a better screening and adequate management of this personality structure in order to allow for a better prevention of suicidal behaviours in this particular subpopulation.

## Figures and Tables

**Table 1:** Sample Description (*n* = 318).

Variables	Categories	%	Major Depression without Suicidal Ideation	Major Depression with Suicidal Ideation	Cramér’s V	*p*-Value
						Chi^2^
Gender	Female (*n* = 182)	57.20%	56.10%	59.00%	−0.0284	0.612
	Male (*n* = 136)	42.80%	43.90%	41.00%		
BMI (kg/m^2^)	≥18 and <25 (*n* = 94)	29.60%	30.10%	28.70%	0.0552	0.616
	≥25 and <30 (*n* = 107)	33.60%	31.60%	36.90%		
	≥30 (*n* = 117)	36.80%	38.30%	34.40%		
Age (years)	30–45 (*n* = 125)	39.30%	45.40%	29.50%	0.1587	0.018
	<30 (*n* = 62)	19.50%	17.90%	22.10%		
	>45 (*n* = 131)	41.20%	36.70%	48.40%		
Benzodiazepine receptor agonists	No (*n* = 251)	78.90%	84.20%	70.50%	0.1633	0.004
	Yes (*n* = 67)	21.10%	15.80%	29.50%		
Antidepressant therapy	No (*n* = 220)	69.20%	76.50%	57.40%	0.2017	<0.001
	Yes (*n* = 98)	30.80%	23.50%	42.60%		
Other psychotropic treatments	No (*n* = 265)	83.30%	84.70%	81.20%	0.0463	0.409
	Yes (*n* = 53)	16.70%	15.30%	18.80%		
Smoking	No (*n* = 245)	77.00%	79.10%	73.80%	0.0614	0.273
	Yes (*n* = 73)	23.00%	20.90%	26.20%		
Alcohol	No (*n* = 167)	52.50%	53.60%	50.80%	0.0268	0.633
	Yes (*n* = 151)	47.50%	46.40%	49.20%		
Somatic treatments	No (*n* = 161)	50.60%	50.50%	50.80%	−0.0030	0.957
	Yes (*n* = 157)	49.40%	49.50%	49.20%		
OSAS	No (*n* = 183)	57.50%	57.10%	58.20%	0.028	0.883
	Mild (*n* = 68)	21.40%	20.90%	22.10%		
	Moderate to severe (*n* = 67)	21.10%	22.00%	19.70%		
Sleep duration (hours)	≥6 (*n* = 209)	65.70%	63.80%	68.90%	−0.0520	0.354
	<6 (*n* = 109)	34.30%	36.20%	31.10%		
Sleep movement disorders	No (*n* = 245)	77.00%	80.10%	72.10%	0.0922	0.1
	Yes (*n* = 73)	23.00%	19.90%	27.90%		
Excessive daytime sleepiness	No (*n* = 143)	45.00%	42.90%	48.40%	−0.0538	0.337
	Yes (*n* = 175)	55.00%	57.10%	51.60%		
Insomnia Severity Index	<15 (*n* = 89)	28.00%	29.10%	26.20%	0.1182	0.109
	≥15 & <22 (*n* = 169)	53.10%	49.00%	59.80%		
	≥22 (*n* = 60)	18.90%	21.90%	14.00%		
Anxiety symptoms	No (*n* = 124)	39.00%	48.50%	23.80%	0.292	<0.001
	Trait anxiety alone (*n* = 40)	12.60%	10.70%	15.60%		
	State anxiety alone (*n* = 52)	16.40%	17.80%	13.90%		
	Trait + state anxiety (*n* = 102)	32.00%	23.00%	46.70%		
BDI (20 items)	<21 (*n* = 172)	54.10%	65.30%	36.10%	0.2853	<0.001
	≥21 (*n* = 146)	45.90%	34.70%	63.90%		
Type D personality	No (*n* = 142)	44.70%	53.60%	30.30%	0.2274	<0.001
	Yes (*n* = 176)	55.30%	46.40%	69.70%		
Suicidal ideation	No (*n* = 196)	61.60%				
	Yes (*n* = 122)	38.40%				
	Median (P25–P75)				Wilcoxon Effect Size (r)	Wilcoxon Test
BMI (kg/m^2^)	27.8 (24.0–32.4)		28.1 (23.9–33.0)	27.6 (24.2–31.0)	0.05	0.372
Age (years)	42 (32–52)		42 (33–51)	44 (31–54)	−0.0400	0.476
ESS	11 (7–14)		11 (7–14)	11 (7–14)	0.0335	0.551
BDI	20 (16–27)		18 (16–22)	25 (19–33)	−0.3526	<0.001
BDI (20 items)	20 (16–26)		18 (16–22)	24 (18–32)	−0.2859	<0.001
ISI	17 (14–21)		17 (13–21)	18 (14–20)	−0.0026	0.964
Spielberger Anxiety Inventory—Trait	51 (45–58)		49 (43–55)	56 (49–62)	−0.3203	<0.001
Spielberger Anxiety Inventory—State	45 (36–54)		42 (34–49)	51 (40–59)	−0.2690	<0.001
DS-14	28 (20–35)		23 (18–32)	33 (25–40)	−0.3539	<0.001
DS—Negative affectivity	15 (11–19)		13 (9–17)	18 (15–22)	−0.4022	<0.001
DS—social inhibition	12 (6–18)		12 (6–16)	16 (9–21)	−0.1939	0.001

BMI = body mass index, OSAS = obstructive sleep apnoea syndrome, BDI = Beck depression inventory, ESS = Epworth sleepiness scale, ISI = insomnia severity index, DS = type-D scale.

**Table 2:** Univariate analyses (*n* = 318).

Variables	Major Depression without Suicidal Ideation	Major Depression with Suicidal Ideation	OR (CI 95%)	*p*-Value
Gender				0.612
Female	60.40%	39.60%	1	
Male	63.20%	36.80%	0.89 (0.56 to 1.40)	
BMI (kg/m^2^)				0.617
<25	62.80%	37.20%	1	
≥25 and <30	57.90%	42.10%	1.22 (0.69 to 2.16)	
≥30	64.10%	35.90%	0.94 (0.54 to 1.66)	
Age (years)				0.019
30–45	71.20%	28.80%	1	
<30	56.50%	43.50%	1.91 (1.01 to 3.60)	
>45	55.00%	45.00%	2.03 (1.21 to 3.40)	
Benzodiazepine receptor agonists				0.004
No	65.70%	34.30%	1	
Yes	46.30%	53.70%	2.23 (1.29 to 3.85)	
Antidepressant therapy				<0.001
No	68.20%	31.80%	1	
Yes	46.90%	53.10%	2.42 (1.49 to 3.95)	
Other psychotropic treatments				0.41
No	62.60%	37.40%	1	
Yes	56.60%	43.40%	1.29 (0.71 to 2.34)	
Smoking				0.274
No	63.30%	36.70%	1	
Yes	56.20%	43.80%	1.34 (0.79 to 2.28)	
Alcohol				0.633
No	62.90%	37.10%	1	
Yes	60.30%	39.70%	1.12 (0.71 to 1.76)	
Somatic treatments				0.957
No	61.50%	38.50%	1	
Yes	61.80%	38.20%	0.99 (0.63 to 1.55)	
OSAS				0.883
No	61.20%	38.80%	1	
Mild	60.30%	39.70%	1.04 (0.59 to 1.84)	
Moderate to severe	64.20%	35.80%	0.88 (0.49 to 1.57)	
Sleep duration (hours)				0.354
≥6	59.80%	40.20%	1	
<6	65.10%	34.90%	0.80 (0.49 to 1.29)	
Sleep movement disorders				0.102
No	64.10%	35.90%	1	
Yes	53.40%	46.60%	1.56 (0.92 to 2.64)	
Excessive daytime sleepiness				0.338
No	58.70%	41.30%	1	
Yes	64.00%	36.00%	0.80 (0.51 to 1.26)	
Insomnia Severity Index				0.112
<15	64.00%	36.00%	1	
≥15 & <22	56.80%	43.20%	1.35 (0.80 to 2.30)	
≥22	71.70%	28.30%	0.70 (0.35 to 1.43)	
Anxiety symptoms				<0.001
No	76.60%	23.40%	1	
Trait anxiety alone	52.50%	47.50%	2.96 (1.40 to 6.26)	
State anxiety alone	67.30%	32.70%	1.59 (0.78 to 3.25)	
Trait + state anxiety	44.10%	55.90%	4.15 (2.35 to 7.34)	
BDI (20 items)				<0.001
<20	74.40%	25.60%	1	
≥21	46.60%	53.40%	3.34 (2.08 to 5.35)	
Type D personality				<0.001
No	73.90%	26.10%	1	
Yes	51.70%	48.30%	2.65 (1.64 to 4.27)	

BMI = body mass index, OSAS = obstructive sleep apnoea syndrome, BDI = Beck depression inventory.

**Table 3:** Multivariate analyses (*n* = 318).

Variables	Model 1 OR Adjusted (CI 95%)	*p*-Value	Model 2 OR Adjusted (CI 95%)	*p*-Value	Model 3 OR Adjusted (CI 95%)	*p*-Value
Type D personality		<0.001		0.036		0.041
No	1		1		1	
Yes	2.41 (1.48 to 3.93)		1.76 (1.04 to 2.97)		1.76 (1.02 to 3.01)	

Model 1 = model adjusted for benzodiazepine receptor agonists and antidepressant therapy. Model 2 = model adjusted for benzodiazepine receptor agonists, antidepressant therapy, anxiety symptoms and depression severity. Model 3 = model adjusted for benzodiazepine receptor agonists, antidepressant therapy, anxiety symptoms, depression severity and age.

**Table 4:** Polysomnographic data (*n* = 318).

	Whole Sample	Major Depression without Suicidal Ideation	Major Depression with Suicidal Ideation	Wilcoxon Effect Size (r)	*p*-Value
Sleep latency (min)	55.3 (30.0–101.5)	55.3 (30.8–103.8)	55.0 (27.5–98.0)	0.0503	0.370
Sleep efficiency (%)	75.3 (65.9–83.0)	74.4 (65.9–83.5)	75.8 (65.5–82.9)	−0.0424	0.450
Sleep period time (min)	439.8 (402.5–473.5)	439.8 (395.5–471.0)	439.8 (409.0–479.0)	−0.0293	0.601
Total sleep time (min)	388.5 (340.0–427.0)	386.5 (332.8–427.8)	390.5 (345.0–426.5)	−0.0292	0.603
% stage 1	7.1 (5.0–9.7)	7.4 (5.2–9.7)	7.0 (4.9–9.6)	0.0461	0.411
% stage 2	49.7 (43.4–57.6)	49.0 (43.1–56.5)	51.2 (43.8–59.8)	−0.1101	0.050
% slow-wave sleep	11.7 (5.3–18.8)	11.7 (5.3–19.5)	11.8 (5.4–17.9)	0.0227	0.686
% REM sleep	16.8 (11.8–21.1)	16.8 (11.8–21.7)	16.9 (12.2–20.3)	0.0607	0.279
REM latency (min)	89.0 (66.0–142.5)	88.3 (62.5–137.0)	93.0 (70.0–155.0)	−0.1085	0.053
% wake after sleep onset	9.5 (5.4–16.4)	9.5 (5.3–16.2)	9.4 (5.7–16.8)	0.0022	0.969
Number of awakenings	22 (16–30)	21 (16–29)	23 (16–31)	−0.0484	0.388
Micro-arousal index	9 (5–15)	9 (5–16)	9 (5–15)	0.0216	0.701
Apnoea–hypopnoea index	3 (1–12)	3 (1–12)	3 (1–12)	−0.0001	0.999
Oxygen desaturation index	3 (0–9)	3 (0–9)	3 (0–10)	−0.0028	0.960
Total time under 90% of SaO_2_ (min)	0.0 (0.0–9.0)	0.0 (0.0–10.3)	0.0 (0.0–7.0)	0.0321	0.567
PLMs index	2 (0–10)	3 (0–9)	2 (0–13)	−0.0117	0.834
	Median (P25–P75)	Median (P25–P75)	Median (P25–P75)		Wilcoxon test

REM = rapid eye movement, PLMs = periodic limb movements during sleep.

## Data Availability

The datasets used and/or analysed during the current study are available from the corresponding author upon reasonable request.

## Supplementary Materials

The following supporting information can be downloaded at: https://www.mdpi.com/article/10.3390/jcm11226611/s1, Annex S1: Detailed description of self-questionnaires used, Annex S2: Description of the sleep assessment, Annex S3: Description of the confounding factors included in the univariate analyses. References [49,50,51,52,53,54,55,56,57,58,59,60,61,62,63,64,65,66,67,68,69,70,71,72,73] were cited in the Supplementary Materials.
