# Supplementary material for: Suicidal Ideation in Major Depressed Individuals: Role of Type D Personality
Source: J Clin Med. 2022 Nov 8;11(22):6611. doi: 10.3390/jcm11226611 (PMC9696109; doi:10.3390/jcm11226611)
Supplement: Supplementary file 1 [file jcm-11-06611-s001.zip › jcm-2002418-supplementary.pdf]

Supplementary Materials

Annex S1

Description of self-questionnaires used:

- The presence of depressive symptoms was investigated using Beck Depression Inventory (BDI-II). This scale consists of 21 items that may be scored from 1 to 3. The score may vary from 0 to 63. A score of 0–9 indicates no depression, 10–18 mild depression, 19–29 moderate depression, and 30–63 severe depression [18].
- Daytime sleepiness was studied using the Epworth Sleepiness Scale. This scale consists of 8 questions that may be scored from 0 to 3 and assesses daytime sleepiness in frequent situations of daily life. The score may vary from 0 to 24. A score greater than 10 indicates excessive daytime sleepiness [19].
- The presence of insomnia symptoms was investigated using the Insomnia Severity Index. This index consists of 7 questions that may be scored from 0 to 4. The score may vary from 0 to 28. A score of 0–7 indicates no insomnia, 8–14 subclinical insomnia, 15–21 moderate insomnia, and 22–28 severe insomnia [20].
- The presence of anxious symptoms was studied using the Spielberger Anxiety Inventory. The state section assesses anxiety during the sleep laboratory whereas the trait section assesses anxiety in daily life. Each of the two sections includes 20 questions graded from 1 to 4. For each section, the score may vary from 20 to 80. The lower the final score, the lower the anxiety level and vice versa [21].

Annex S2

Description of the sleep assessment

In major depressed individuals recruited for this study, a specific semi-structured sleep interview based on the recommendations of the *American Academy of Sleep Medicine* was performed by a unit psychiatrist during their admission to the Sleep Laboratory in order to allow a systematic assessment of their complaints related to sleep including sleeping habits, symptoms of insomnia disorder, symptoms of sleep-related breathing disorders, symptoms of central disorders of hypersomnolence, symptoms of circadian rhythm sleep-wake disorders, symptoms of parasomnias and symptoms of sleep-related movement disorders [23]. This specific semi-structured sleep interview is a standardised procedure of the Sleep Laboratory that makes it possible to systematically research for symptoms suggestive of sleep disorders and to program polysomnographic recordings adapted to the symptoms reported by patients.

During their stay in the Sleep Laboratory, major depressed individuals included in this study benefited from a polysomnographic recording from which the data were collected for analysis. The patients went to bed between 22:00 - 24:00 and got up between 6:00 - 8:00, following their usual schedule. During bedtime hours, the subjects were recumbent and the lights were turned off. Daytime naps were not permitted.

The polysomnographic recordings performed in our unit meet the recommendations of the *American Academy of Sleep Medicine* [49]. The applied polysomnography-montage was as follows: two electro-oculogram channels, three electroencephalogram channels (Fz-Ax, Cz-Ax, and Oz-Ax, where Ax was a A1A2 mastoid reference), one submental electromyogram channel, electrocardiogram, pressure *cannula* to detect the oro-nasal airflow, finger pulse-oximetry, a microphone to record breathing sounds and snoring, plethysmographic inductive belts to measure thoracic and abdominal breathing, and anterior tibialis electrodes. Polysomnographic recordings were visually scored by specialised technicians according to the criteria of the *American Academy of Sleep Medicine* [50].

Obstructive apnoeas were scored if the decrease in air flow was ≥90% for at least 10 seconds whereas obstructive hypopnoeas were scored if the decrease in airflow was

≥30% for at least 10 seconds with a decrease in oxygen saturation of 3% or followed by microarousal [51]. The obstructive apnoea-hypopnoea index correspond to the total number of obstructive apnoeas and hypopnoeas divided by the period of sleep in hours. Obstructive sleep apnoea syndrome was considered absent when the obstructive apnoea-hypopnoea index was <5/h, mild when the obstructive apnoea-hypopnoea index was ≥5/h & <15/h and moderate to severe when the obstructive apnoea-hypopnoea index was ≥15/h [52].

Periodic limb movements during sleep were scored on the basis of the following strict criteria: (1) duration between 0.5 to 10 seconds, (2) interval between 5 and 90 seconds from leg movement onset and (3) movements had to be part of a series of ≥4 consecutive movements meeting these criteria [53]. Periodic limb movement's index corresponds to the total number of periodic limb movements during sleep divided by period of sleep in hours. Moderate to severe periodic limb movements during sleep were considered to be present when the periodic limb movement's index was ≥15/hour [54]. Moreover, the diagnoses of restless legs syndrome were made according to the diagnostic criteria of the *International Restless Legs Syndrome Study Group* [55].

Finally, potential diagnoses of insomnia disorders were made according to the diagnostic criteria of the *American Academy of Sleep Medicine Work Group* [56] whereas short sleep duration was defined as sleep time <6 hours [57].

Annex S3

Description of the confounding factors included in the univariate analyses

After a review of the literature on the factors associated with SI in the general population and in major depressed individuals [58-73], the potential confounding factors included in this study were body mass index (categorised: <25 kg/m², ≥25 & <30 kg/m², ≥30 kg/m²), age (categorised: <30 years, 30-45 years, >45 years), presence of obstructive sleep apnoea syndrome (categorised: absent, mild, moderate to severe), sleep duration (categorised: <6 hours, ≥6 hours), insomnia severity (categorised based on Insomnia Severity Index scores: <15, ≥15 & <22, ≥22), anxiety symptoms (categorised based on Trait–State Spielberger Anxiety Inventory scores: absent, trait anxiety alone, state anxiety alone, trait + state anxiety), depression severity (categorised based on Beck Depression Inventory (BDI-II) scores [without item 9–suicidal ideation]: <21, ≥21) and as binary variables: gender, benzodiazepine receptor agonists, antidepressant therapy, other psychotropic treatments (neuroleptics, thymostabilisers or pain medications), alcohol consumption, smoking, excessive daytime sleepiness, somatic treatments and sleep movement disorders.

References

1. Kushida, C.A.; Littner, M.R.; Morgenthaler, T.; Alessi, C.A.; Bailey, D.; Coleman, J.; Friedman, L.; Hirshkowitz, M.; Kapen, S.; Kramer, M.; Lee-Chiong, T.; Loube, D.L.; Owens, J.; Pancer, J.P.; Wise; M. Practice parameters for the indications for polysomnography and related procedures: an update for 2005. *Sleep* **2005**, *28*, 499–521.
2. Iber, C.; Ancoli-Israel, S.; Chesson, A.; Quan SF for the American Academy of Sleep Medicine. *The AASM Manual for the Scoring of Sleep and Associated Events: Rules, Terminology and Technical Specifications*, 1st. ed.; American Academy of Sleep Medicine: Westchester, IL, USA, 2007.
3. Berry, R.B.; Budhiraja, R.; Gottlieb, D.J.; Gozal, D.; Iber, C.; Kapur, V.K.; Marcus, C.L.; Mehra, R.; Parthasarathy, S.; Quan, S.F.; Redline, S.; Strohl, K.P.; Davidson Ward, S.L.; Tangredi, M.M.; American Academy of Sleep Medicine. Rules for scoring respiratory events in sleep: update of the 2007 AASM Manual for the Scoring of Sleep and Associated Events. Deliberations of the Sleep Apnea Definitions Task Force of the American Academy of Sleep Medicine. *J. Clin. Sleep Med.* **2012**, *8*, 597–619.
4. Fleetham, J.; Ayas, N.; Bradley, D.; Ferguson, K.; Fitzpatrick, M.; George, C.; Hanly, P.; Hill, F.; Kimoff, J.; Kryger, M.; Morrison, D.; Series, F.; Tsai, W.; CTS Sleep Disordered Breathing Committee. Canadian Thoracic Society guidelines: diagnosis and treatment of sleep disordered breathing in adults. *Can. Respir. J.* **2006**, *13*, 387–392.
5. Ferri, R.; Koo, B.B.; Picchietti, D.L.; Fulda, S. Periodic leg movements during sleep: phenotype, neurophysiology, and clinical significance. *Sleep Med.* **2017**, *31*, 29–38.
6. Haba-Rubio, J.; Marti-Soler, H.; Tobback, N.; Andries, D.; Marques-Vidal, P.; Vollenweider, P.; Preisig, M.; Heinzer, R. Clinical significance of periodic limb movements during sleep: the HypnoLaus study. *Sleep Med.* **2018**, *41*, 45–50.

7. Allen, R.P.; Picchietti, D.L.; Garcia-Borreguero, D.; Ondo, W.G.; Walters, A.S.; Winkelman, J.W.; Zucconi, M.; Ferri, R.; Trenkwalder, C.; Lee, H.B.; International Restless Legs Syndrome Study Group. Restless legs syndrome/Willis-Ekbom disease diagnostic criteria: updated International Restless Legs Syndrome Study Group (IRLSSG) consensus criteria--history, rationale, description, and significance. *Sleep Med*. **2014**, *15*, 860–873.
8. Edinger, J.D.; Bonnet, M.H.; Bootzin, R.R.; Doghramji, K.; Dorsey, C.M.; Espie, C.A.; Jamieson, A.O.; McCall, W.V.; Morin, C.M.; Stepanski, E.J; American Academy of Sleep Medicine Work Group. Derivation of research diagnostic criteria for insomnia: report of an American Academy of Sleep Medicine Work Group. *Sleep* **2004**, *27*, 1567–1596.
9. Hein, M.; Lanquart, J.P.; Loas, G.; Hubain, P.; Linkowski, P. *Insomnia with short sleep duration as risk factor for type 2 diabetes: a systematic review of the literature.* Rev. Med. Brux. **2020**, 41, 98–104.
10. Amiri, S.; Behnezhad, S. Body mass index and risk of suicide: A systematic review and meta-analysis. *J. Affect. Disord*. **2018**, *238*, 615–625.
11. Aaltonen, K.; Näätänen, P.; Heikkinen, M.; Koivisto, M.; Baryshnikov, I.; Karpov, B.; Oksanen, J.; Melartin, T.; Suominen, K.; Joffe, G.; Paunio, T.; Isometsä, E. Differences and similarities of risk factors for suicidal ideation and attempts among patients with depressive or bipolar disorders. *J. Affect. Disord*. **2016**, *193*, 318–330.
12. Shen, Y.; Meng, F.; Tan, S.N.; Zhang, Y.; Anderiescu, E.C.; Abeysekera, R.E.; Luo, X.; Zhang, X.Y. Excessive daytime sleepiness in medical students of Hunan province: Prevalence, correlates, and its relationship with suicidal behaviors. *J. Affect. Disord*. **2019**, *255*, 90–95.
13. Kivelä, L.; Krause-Utz, A.; Mouthaan, J.; Schoorl, M.; de Kleine, R.; Elzinga, B.; Eikelenboom, M.; Penninx, B.W.; van der Does, W.; Antypa, N. Longitudinal course of suicidal ideation and predictors of its persistence - A NESDA study. *J. Affect. Disord*. **2019**, *257*, 365–375.
14. Kavalidou, K.; Smith, D.J.; O'Connor, R.C. The role of physical and mental health multimorbidity in suicidal ideation. *J. Affect. Disord*. **2017**, *209*, 80–85.
15. Timkova, V.; Nagyova, I.; Reijneveld, S.A.; Tkacova, R.; Stewart, R.E.; van Dijk, J.P.; Bültmann, U. Suicidal ideation in patients with obstructive sleep apnoea and its relationship with disease severity, sleep-related problems and social support. *J. Health Psychol*. **2020**, *25*, 1450–1461.
16. Para, K.S.; Chow, C.A.; Nalamada, K.; Kakade, V.M.; Chilakamarri, P.; Louis, E.D.; Koo, B.B. Suicidal thought and behavior in individuals with restless legs syndrome. *Sleep Med*. **2019**, *54*, 1–7.
17. Littlewood, D.L.; Kyle, S.D.; Carter, L.A.; Peters, S.; Pratt, D.; Gooding, P. Short sleep duration and poor sleep quality predict next-day suicidal ideation: an ecological momentary assessment study. *Psychol. Med*. **2019**, *49*, 403–411.
18. Loas, G.; Dalleau, E.; Lecointe, H.; Yon, V. Relationships between anhedonia, alexithymia, impulsivity, suicidal ideation, recent suicide attempt, C-reactive protein and serum lipid levels among 122 inpatients with mood or anxious disorders. *Psychiatry Res*. **2016**, *246*, 296–302.
19. Kim, K.M.; Hwang, H.R.; Kim, Y.J.; Lee, J.G.; Yi, Y.H.; Tak, Y.J.; Lee, S.H.; Chung, S.I. Association between Serum-Ferritin Levels and Sleep Duration, Stress, Depression, and Suicidal Ideation in Older Koreans: Fifth Korea National Health and Nutrition Examination Survey 2010–2012. *Korean J. Fam. Med*. **2019**, *40*, 380–387.
20. Stübner, S.; Grohmann, R.; Greil, W.; Zhang, X.; Müller-Oerlinghausen, B.; Bleich, S.; Rüther, E.; Möller, H.J.; Engel, R.; Falkai, P.; Toto, S.; Kasper, S.; Neyazi, A. Suicidal Ideation and Suicidal Behavior as Rare Adverse Events of Antidepressant Medication: Current Report from the AMSP Multicenter Drug Safety Surveillance Project. *Int. J. Neuropsychopharmacol*. **2018**, *21*, 814–821.
21. Pigeon, W.R.; Woosley, J.A.; Lichstein, K.L. Insomnia and hypnotic medications are associated with suicidal ideation in a community population. *Arch. Suicide Res*. **2014**, *18*, 170–180.
22. Gallyer, A.J.; Dougherty, S.P.; Gai, A.R.; Stanley, I.H.; Hom, M.A.; Rogers, M.L.; Duffy, M.E.; Buchman-Schmitt, J.M.; Spencer-Thomas, S.; Joiner, T.E. Problematic alcohol use and suicidal ideation among firefighters: A multi-study investigation of the explanatory roles of perceived burdensomeness and thwarted belongingness. *J. Affect. Disord*. **2018**, *238*, 281–288.
23. Park, H.; Suh, B.S.; Lee, K. Relationship between daily coffee intake and suicidal ideation. *J. Affect. Disord*. **2019**, *256*, 468–472.
24. Hintikka, J.; Koivumaa-Honkanen, H.; Lehto, S.M.; Tolmunen, T.; Honkalampi, K.; Haatainen, K.; Viinamäki, H. Are factors associated with suicidal ideation true risk factors? A 3-year prospective follow-up study in a general population. *Soc. Psychiatry Psychiatr. Epidemiol*. **2009**, *44*, 29–33.
25. Reutfors, J.; Andersson, T.M.; Tanskanen, A.; DiBernardo, A.; Li, G.; Brandt, L.; Brenner, P. Risk Factors for Suicide and Suicide Attempts Among Patients with Treatment-Resistant Depression: Nested Case-Control Study. *Arch. Suicide Res*. **2021**, *25*, 424–438.
